# Understanding Conversation between Older Adults: An Observational Study

**DOI:** 10.1093/geroni/igaf122.4224

**Published:** 2025-12-31

**Authors:** Fuxi Ouyang, W Quin Yow

**Affiliations:** Singapore University of Technology and Design, Singapore, Singapore; Singapore University of Technology and Design, Singapore, Singapore

## Abstract

Previous studies have shown older adults’ conversations featuring reminiscence, elaboration, multimodal expression, and reduced cohesion. However, most research focuses on monolingual populations, with limited attention to multilingual environments. This gap is important because multilingualism shapes cognitive, social, and communicative processes in distinct ways. As part of a larger research project, we conducted an observation study to understand the characteristics of bilingual older adults’ conversations in Singapore. Twenty older adults (mean-age=75.6; 19F) were randomly paired and asked to engage in an unstructured conversation with their partner for 20 to 30 minutes. This approach, while situated in a study setting, was intended to elicit spontaneous conversational behavior that approximates naturalistic communication. Conversations were audio-recorded, and two coders applied global topic coding to the transcripts. These conversations were analysed for the frequency and commonality of topics and vocabulary in the conversations. Six categories were found to be frequently referenced (decreasing-order): Community Centre, Recreation, Shopping, Food, Family, and Travel. Analyses also revealed that a single topic typically dominated the discussion, accounting for more than 50% of all segments. In addition, based on the five-person-frame-reference, Self (mean=37.5%), None–Inanimate (mean=20.7%), and Other Speaker (mean=19.5%) were the most frequently referenced. References to Relatives (mean=7.6%) and Service Providers (mean=4.0%) were relatively rare. For time frame references, we found that most conversations were focused on the Present (mean=56.2%) and the Past (mean=33.9%), followed by Future-oriented content (mean=9.9%). These results demonstrated conversational characteristics among older adults in multilingual contexts, contributing to a deeper understanding of communicative patterns in diverse societies.

